# A Pilot Study: Warm Stimulation on Guangming (GB37) to Relief Asthenopia

**DOI:** 10.1155/2015/641792

**Published:** 2015-05-03

**Authors:** Tao Huang

**Affiliations:** Institute of Acupuncture and Moxibustion, China Academy of Chinese Medical Sciences, Beijing 100700, China

## Abstract

Infrared thermometry was performed in 15 female asthenopia patients (average ± SD: 54.88 ± 7.30 years) prior to, during, and after stimulation using electrothermal Bian-stone at the Guangming (GB37) acupoints. The results of this controlled pilot study (control points at the Yongquan (KI1) and Tianshu (ST25) points) showed significant (*P* ≤ 0.05) increases in eyes' temperature. At the same time, no changes were found at the control points. Furthermore, after warm stimulation on Guangming (GB37) acupoints, the clinical symptoms were getting better than the control points. The symptoms' score was decreased significantly too (*P* ≤ 0.05). It was demonstrated that there is some relationship between Guangming (GB37) point and eyes, and warm stimulation on Guangming (GB37) could relief uncomfortable of asthenopia.

## 1. Introduction

Asthenopia is very common seen in later-middle age women and with the symptoms like eye fatigue, ache, dryness and heaviness in the eye, itching, red eyes, blurred vision, tearing, and so on. In TCM textbook, there are report that acupuncture Guangming (GB37) could cure “blue blind” and could adjust liver and gallbladder meridian [1].

In the former studies, electrothermal Bian-stone was used to observe the influence of blood flux of brain by warm stimulation on local points and could increase the flux of blood at local area [2, 3]. The goal of this experiment is to investigate the clinical effects and change of eyes' temperature on the asthenopia patients to find out the interrelationship between eyes and distant acupoints.

## 2. Materials and Methods

### 2.1. Electrothermal Bian-Stone

**Figure 1:**
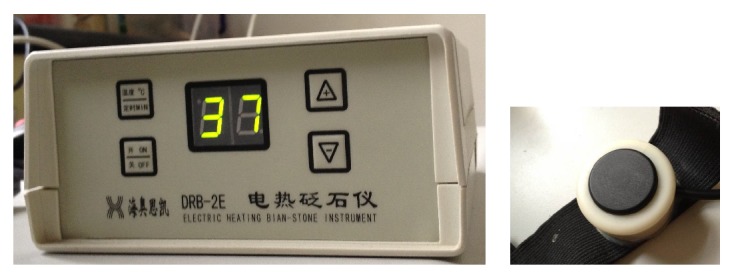
Electrothermal Bian-stone instrument and its stone probe.

The electrothermal Bian-stone apparatus is made by using a special Sibinfu stone with the ultrasound, far-infrared, and microcrystal properties and by using the modern electrothermal and microcomputer temperature control technology [4]. It was proved to have the similar function as traditional moxibustion [5]. Compared with traditional moxibustion, it can offer adjustable and constant temperature and without the pollution of moxa smoking and noxious odour. DRB-2E electrothermal Bian-stone moxibustion apparatus is produced by Beijing Healthcare Company, China, with 2 small probes which could be fixed on the acupoint. The temperature of probe was chosen 37°C (see Figure 1). The treatment time was 30 minutes.

### 2.2. Asthenopia Patients' Inclusion Criteria

15 asthenopia patients (mean ± SD: age 54.88 ± 7.30 years; range 45–65 years; all female) were studied. All of them fill in the asthenopia questionnaire including 8 common clinical symptoms like dryness, foreign body sensation, itching, and heaviness in the eye, tearing, redness, blurred vision, and photophobia. They were informed of the nature of the investigation as far as the study design allowed. The study was approved by the local ethics committee and all persons gave written informed consent.

### 2.3. Measuring of Eye's Temperature Using FLIR i7

**Figure 2:**
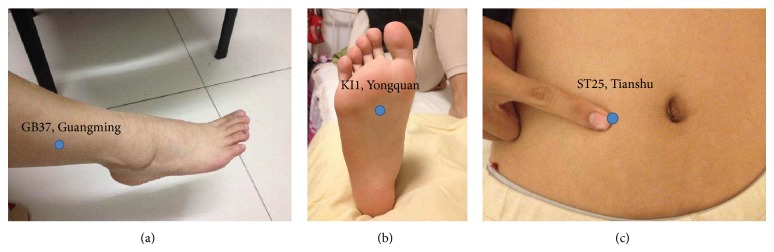
(a) Location of Guangming (GB37): 5 cun upper the prominence of the medial malleolus. (b) Location of Yongquan (KI1): 1/3 forth of fossa of foot. (c) Location of Tianshu (ST25): 2 cun beside the navel.

Each patient accepted 2 times treatments and measurements in different day. Room temperature was constant at 26°C. Participants lay face up on a bed with DRB-2E electrothermal Bian-stone (Healthcare Company, Beijing, China) probes on both Guangming (GB37) acupoints, Yongquan (KI), or Tianshu (ST25) as the control points at random (see Figures 2(a)–2(c)). Using the thermal infrared imager FLIR i7 (FLIR Company, Portland, USA) measured the patients' eyes before, during 10 to 30 minutes, and 10 minutes after removing the stone probes.

### 2.4. Statistical Analysis

Data of temperature of eyes were transferred into measurement by FlIR QuickReport 1.2 SP1 (FLIR company, Portland, USA). Symptom score and the measurement data were all analyzed using Friedman repeated measures ANOVA on ranks and Tukey's test was used for analysis post hoc. The level of significance was defined as *P* < 0.05.

## 3. Results

### 3.1. Change of the Eyes' Temperature

The eyes' temperature of Guangming (GB37) increased significantly after accepting the warm stimulation and decreased significantly after removing the warm stone probes too. But in control group, neither matter Yongquan (KI1) nor Tianshu (ST25), the change of eyes' temperature was not significant (see Table 1).

**Figure 3:**
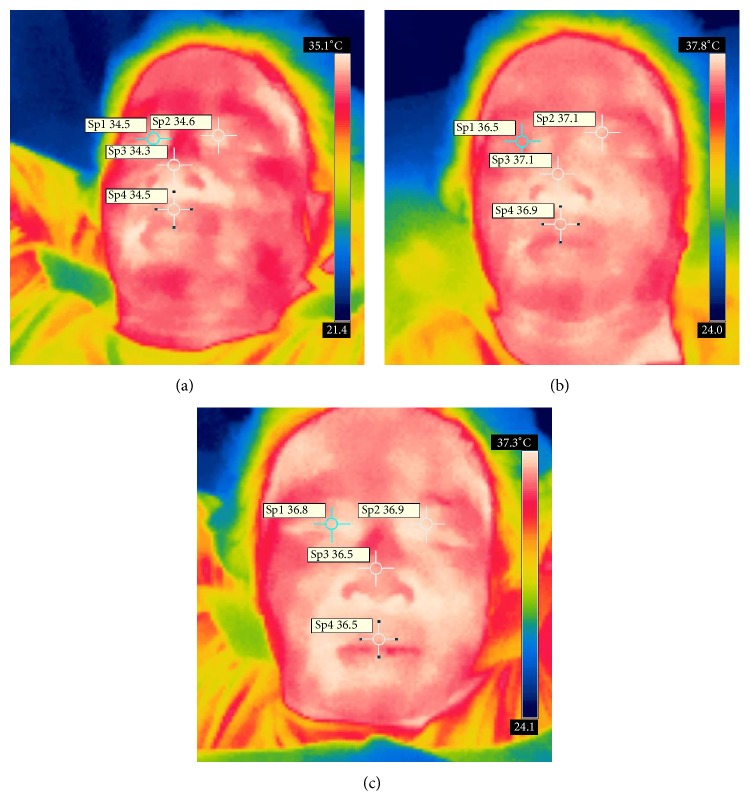
The change of eyes' temperature, before, during, and 10 minutes after removing the warm stimulation on Guangming (GB37).

**Figure 4:**
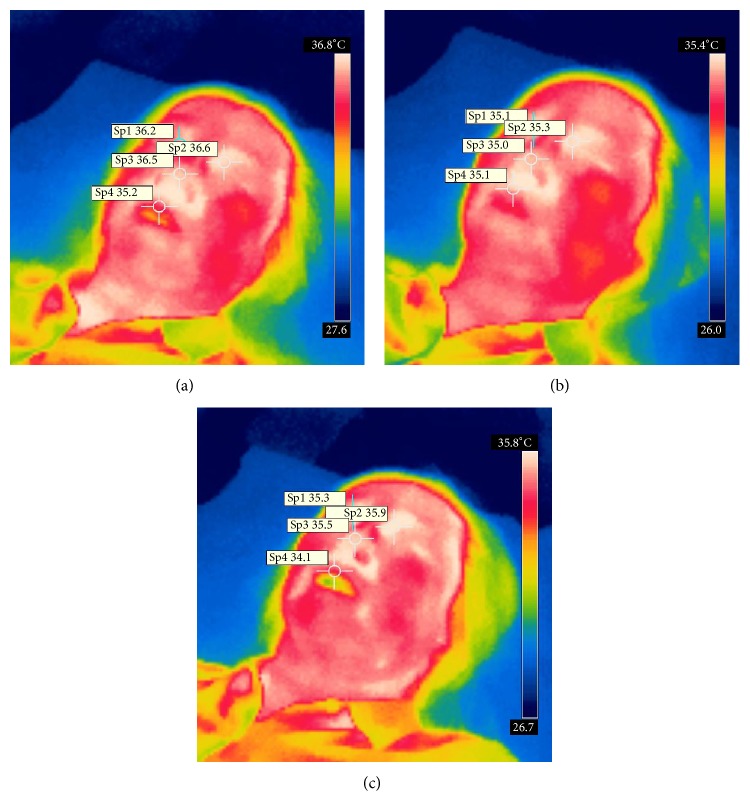
The change of eyes' temperature, before, during, and 10 minutes after removing the warm stimulation on control point Yongquan (KI1).

Figures 3(a)–3(c) and Figures 4(a)–4(c) displayed the representative images.

### 3.2. The Improvement of Clinical Symptoms

Almost patients reported that the symptoms like dryness, itching, and blurred vision got better and felt bright eyed immediately. But the redness had no change. Although the long-term therapeutic effect cannot be proved, the symptoms score was also improved (see Table 2).

## 4. Discussion

As a high-tech moxibustion instrument, electrothermal Bian-stone could offer adjustable and constant temperature, with the effects of warming and activating the channels, dispelling cold, and promoting blood flow.

Guangming (GB37) is Luo-connecting point of gallbladder meridian/channel and connects liver and GB meridian/channel. In the earliest acupuncture literature* Classic of Acupuncture and Moxibustion (Zhenjiujiayijing)*, there was not any record about using Guangming (GB37) to treat eye's diseases [6]. But some scholar of later period thought that it could cure eye diseases including glaucoma and cataract [7]. Because liver opens into eyes, it seem as make sense in TCM. What is the relationship between Guangming (GB37) and eyes? This experiment attempts to answer this question. Warm stimulation given on Guangming (GB37) could increase the temperature of patients' eye significantly and improved the clinical symptoms in short time. Same stimulation on control points had no effect.

## 5. Conclusion

Acupoint Guangming (GB37) could specifically work in the eyes, and the function channel might be Chinese liver and gallbladder channels. Warm stimulation on Guangming (GB37) could relief the asthenopia. The correct and long term effect is worth further study.

## Figures and Tables

**Table 1:** The change of eyes' temperature before, during, and after warm stimulation (*x* ± *s*).

	The temperature of both eyes (°C), *N* = 15	
	Guangming (GB37)	Control points
Before treatment	35.72 ± 0.70	36.04 ± 0.59
During treatment	36.52 ± 0.34^*^	36.12 ± 0.47
10 mins after removing the stone probes	36.30 ± 0.37^*^	36.05 ± 0.55

^*^ *P*≤ 0.05.

**Table 2:** The change of asthenopia symptoms score (*x* ± *s*).

	The change of symptom score, *N* = 15	
	Guangming (GB37)	Control points
Before treatment	59.67 ± 12.50	57.04 ± 8.90
After treatment	35.67 ± 6.51^**^	54.67 ± 10.02

^**^ *P* < 0.01.
